# Testing Seeds

**Published:** 1881-10

**Authors:** 


### TESTING SEEDS.

The following is a brief extract of a paper by Professor W. S. Beal, presented at a meeting of the Association for the Promotion of Agricultural Science, held August 16, in Cincinnati, Ohio.

Good fresh seeds varied much less in the per cent. which germinated than did those which possessed little or low vitality. With the exception of two kernels in two different lots of fresh hand-picked wheat, 100 per cent. germinated in all the tests made, excepting those in open ground, where 94.9 per cent. out of 1,000 kernels germinated.

Some poorer old wheat, the history of which was not known, when tested in the same manner as the new wheat, and at the same time varied from 39 to 86.8 per cent. in germination. Red wheat germinated more slowly than white wheat.

Wheat was once germinated and well dried in the sun. Quite a large per cent. germinated a second time, depending on how far the process had gone before it was checked by drying. Of this well dried grain a considerable portion germinated the second time.

Seeds of pumpkins and the larger squashes, tested at 80 degrees Fahr., or lower, showed variable and unsatisfactory results. While tested at 100 degrees to 136 degrees the per cent. in germination was much higher and more uniform.

In all the above cases seeds were tested in lots of 50 or 100 seeds. They were tested in porous saucers kept damp, in damp sand, in soil in the garden, and in folds of damp paper.—*Scientific American.*

